# Resveratrol Protects C6 Astrocyte Cell Line against Hydrogen Peroxide-Induced Oxidative Stress through Heme Oxygenase 1

**DOI:** 10.1371/journal.pone.0064372

**Published:** 2013-05-15

**Authors:** André Quincozes-Santos, Larissa Daniele Bobermin, Alexandra Latini, Moacir Wajner, Diogo Onofre Souza, Carlos-Alberto Gonçalves, Carmem Gottfried

**Affiliations:** 1 Departamento de Bioquímica, Programa de Pós-Graduação em Ciências Biológicas: Bioquímica, Instituto de Ciências Básicas da Saúde, Universidade Federal do Rio Grande do Sul, Porto Alegre, Rio Grande do Sul, Brazil; 2 Departamento de Bioquímica, Centro de Ciências Biológicas, Universidade Federal de Santa Catarina, Florianópolis, Santa Catarina, Brazil; Massachusetts General Hospital/Harvard Medical School, United States of America

## Abstract

Resveratrol, a polyphenol presents in grapes and wine, displays antioxidant and anti-inflammatory properties and cytoprotective effect in brain pathologies associated to oxidative stress and neurodegeneration. In previous work, we demonstrated that resveratrol exerts neuroglial modulation, improving glial functions, mainly related to glutamate metabolism. Astrocytes are a major class of glial cells and regulate neurotransmitter systems, synaptic processing, energy metabolism and defense against oxidative stress. This study sought to determine the protective effect of resveratrol against hydrogen peroxide (H_2_O_2_)-induced cytotoxicity in C6 astrocyte cell line, an astrocytic lineage, on neurochemical parameters and their cellular and biochemical mechanisms. H_2_O_2_ exposure increased oxidative-nitrosative stress, iNOS expression, cytokine proinflammatory release (TNFα levels) and mitochondrial membrane potential dysfunction and decreased antioxidant defenses, such as SOD, CAT and creatine kinase activity. Resveratrol strongly prevented C6 cells from H_2_O_2_-induced toxicity by modulating glial, oxidative and inflammatory responses. Resveratrol *per se* increased heme oxygenase 1 (HO1) expression and extracellular GSH content. In addition, HO1 signaling pathway is involved in the protective effect of resveratrol against H_2_O_2_-induced oxidative damage in astroglial cells. Taken together, these results show that resveratrol represents an important mechanism for protection of glial cells against oxidative stress.

## Introduction

The polyphenol resveratrol (3,5,4′-trihydroxy-trans-stilbene), a redox active compound, is a phytoalexin found in a wide variety of dietary sources including grapes, peanuts and wines, especially red wines [1], [2], [3], [4], [5]. Resveratrol presents important antioxidant properties, possibly by its direct scavenging effect and/or activation of pathways those upregulate natural antioxidant defenses [6], [7]. Many studies have shown that resveratrol can prevent or slow the progression of a wide variety of illnesses, including cancer [8] and cardiovascular diseases [9], [10] and improves health and survival of mice on a high-calorie diet [11]. Moreover, it has been demonstrated that resveratrol has beneficial effects in neurological diseases [6], [12], [13], [14] and is able to inhibit β-amyloid peptide neurotoxicity [15], [16]. Whilst direct protective effects of resveratrol against oxidative stress have been demonstrated in neuroglial cells [14], [17], [18], [19], [20], [21], [22], the mechanisms of these effects are not fully understood.

Reactive oxygen species (ROS), such as hydrogen peroxide (H_2_O_2_), are generated during normal cellular metabolism, playing important roles in signaling pathways [23], [24]. However, H_2_O_2_ also presents toxicological effects, because it can produce new radicals and induces damage to major cellular constituents [25], [26], [27]. Moreover, it has been demonstrated that exogenous H_2_O_2_ promotes an imbalance between production and removal of ROS towards the pro-oxidative state, often referred to as oxidative stress.

Heme oxygenase (HO) is the rate-limiting enzyme in the degradation pathway of pro-oxidant heme into biliverdin/bilirubin (both known antioxidants) and has two isoforms: the inducible HO1 and the constitutive HO2 [28]. In the central nervous system (CNS), HO pathway has been reported to be active and to operate as a fundamental defensive mechanism for cells exposed to an oxidant challenge [29]. Increases in HO1 protein levels are associated to protection against stress conditions, such as oxidative stress and hypoxia [30]. HO1 is able to inhibit inducible nitric oxide synthase (iNOS) activity [31], an isoform of nitric oxide synthase that catalyze the synthesis of nitric oxide (NO) from L-arginine. NO plays a physiological role in neuronal cell signaling, but, when in elevated levels, NO becomes noxious, can interact with superoxide anion (generated by mitochondria or by other mechanisms) leading to the overproduction of powerful oxidant species peroxynitrite [32]. This toxic compound belongs to a family known as reactive nitrogen species or RNS, which cause cellular damage [33], [34]. It has been frequently suggested that polyphenols help to regulate the ROS/RNS imbalance [29], [35], [36] with potential effect on neuroglial plasticity modulation, including astrocytes activity regulation [14].

Astrocytes have been shown to be involved in the regulation of the brain microenvironment, in particular regarding neurotransmitter systems and ionic homeostasis, synaptic transmission, metabolic support, free-radical scavenging, maintenance of the blood-brain barrier and immune function [37], [38], [39]. Astrocytes express numerous receptors that enable them to respond to various neuroactive compounds, including neurotransmitters, neuropeptides, growth factors, cytokines, small molecules and toxins [40]. The C6 astrocyte cell line [98% GFAP (glial fibrillary acidic protein) positive after 100 passages] are widely used as an astrocyte-like cell line to study astrocytic function, *e.g.* glutamate uptake, glutamine synthetase activity, S100B secretion and parameters of oxidative stress. Moreover, this cell line responds quickly to external stimuli, such as H_2_O_2_, which can generate oxidative-nitrosative stress [17], [41].

Recently, our group has reported that resveratrol was able to modulate important glial parameters involved in brain plasticity and prevents lipid peroxidation, DNA damage and genotoxicity induced by H_2_O_2_ exposure in astroglial cells [14], [17], [41]. Thus, in the present study, we investigated the effect of resveratrol against H_2_O_2_-induced toxicity in C6 astrocyte cell line on neurochemical parameters and their cellular and biochemical mechanisms. Nitrite production; the expression of iNOS and HO1 proteins; intracellular ROS production; total antioxidant reactivity (TAR) levels; mitochondrial membrane potential and creatine kinase (CK) activity were assessed. The activity of the main antioxidant enzymes: superoxide dismutase (SOD – EC 1.15.1.1), catalase (CAT – EC 1.11.1.6) and glutathione peroxidase (GPx – EC 1.11.1.9); the extracellular glutathione (GSH) levels and tumor necrosis factor α (TNFα) levels were also evaluated. Additionally, the mechanism of the protective effect of resveratrol against H_2_O_2_-oxidative insult was also explored.

## Materials and Methods

### Materials

Resveratrol, chemical reagents, anti-iNOS and cell culture materials were purchased from Sigma (St. Louis, MO, USA), except for Dulbecco’s Eagle’s medium (DMEM), fetal bovine serum (FBS) and JC-1, which were purchased from Gibco/Invitrogen (Carlsbad, CA, USA). Anti-HO1 was obtained from Santa Cruz Biotechnology (Santa Cruz, CA, USA). RANSOD was purchased from Randox (Crumlin, CA, UK). All other chemicals were purchased from local commercial suppliers.

### Maintenance of Cell Culture

The C6 astrocyte cell line were obtained from the American Type Culture Collection (Rockville, MD, USA) and were maintained essentially according to previously described [42]. The cells were seeded in flasks and cultured in DMEM (pH 7.4) containing 5% FBS, 2.5 mg/mL Fungizone® and 100 U/l gentamicin. Cells were kept at a temperature of 37°C in an atmosphere of 5% CO_2_/95% air. Exponentially growing cells were detached from the culture flasks using 0.05% trypsin/ethylene-diaminetetracetic acid (EDTA) and seeded 10×10^3^ cells/cm^2^ in 96-, 24- or 6-well plates.

### Resveratrol and Hydrogen Peroxide Treatment

After cells reached confluence, the culture medium was removed from well plates and cells were pre-incubated in the absence or presence of 100 µM of resveratrol for 1 h, in serum-free DMEM (pH 7.4). After pre-incubation, resveratrol was maintained and 1 mM H_2_O_2_ was added for 0.5 h. During all procedure, cells were maintained at 37°C in an atmosphere of 5% CO_2_/95% air. Control cells were exposed to 0.25% ethanol vehicle. For all parameters analyzed, the results obtained with vehicle were not different from those obtained under basal conditions without ethanol.

### Nitric Oxide Production

Nitric oxide was determined by measurement of nitrite (a stable oxidation product of NO), based on the Griess reaction. The Griess reagent was prepared by mixing equal volumes of 1% sulfanilamide in 0.5 M HCl and 0.1% N-(1-naphthyl) ethylenediamine in deionized water. The assay was performed as described [43], with modifications. Briefly, cells were cultured on 96-well plate and after treatment, the Griess reagent was added directly to the cell culture and the incubation was maintained under reduced light at room temperature during 15 min. Samples were analyzed at 550 nm on a microplate spectrophotometer. Controls and blanks were run simultaneously. Nitrite concentrations were calculated using a standard curve prepared with sodium nitrite (0–50 µM).

### Western Blot Analysis

Cells were removed from plates after treatments using lysis solution with 4% SDS, 2 mM EDTA, 50 mM Tris-HCl, pH 6.8. Equal amounts of proteins from each sample were boiled in sample buffer [62.5 mM Tris-HCl, pH 6.8, 2% (w/v) SDS, 5% β-mercaptoethanol, 10% (v/v) glycerol, 0.002% (w/v) bromophenol blue] and submitted to electrophoresis in 10% (w/v) SDS-polyacrylamide gel. The separated proteins were blotted onto a nitrocellulose membrane. Equal loading of each sample was confirmed with Ponceau S staining (Sigma). The following polyclonal antibodies were used: anti-iNOS (1∶10000; Sigma) and anti-HO1 (1∶3000; Santa Cruz). β-actin was used as a loading control. After incubating overnight with the primary antibody at room temperature, membranes were washed and incubated with peroxidase-conjugated anti-rabbit immunoglobulin (IgG) at a dilution of 1∶1000 for 1 h. The chemiluminescence signal was detected using an ECL (Amersham), after the films were scanned and bands were quantified using the Scion Image software.

### Intracellular ROS Production

DCFH oxidation was used to measure intracellular ROS production. DCFH-DA (2′-7′-dichlorofluorescein diacetate) is hydrolyzed by intracellular esterases to dichlorofluorescin (DCFH), which is trapped within the cell. This non-fluorescent molecule is then oxidized to fluorescent dichlorofluorescin (DCF) by action of cellular oxidants. To test whether HO1 was involved in the effect of resveratrol against H_2_O_2_-induced increase ROS production, we used its specific inhibitor, Zinc Protoporphyrin IX (ZnPP IX) 10 µM, for 0.5 h before the treatment described for resveratrol and H_2_O_2_. *After,* cells were treated with DCFH-DA (10 µM) for 0.5 h at 37°C. Following DCFH-DA exposure, the cells were scraped into PBS with 0.2% Triton X-100. The fluorescence was measured in a plate reader (Spectra Max GEMINI XPS, Molecular Devices, USA) with excitation at 485 nm and emission at 520 nm [17]. The ROS production was calculated as Unit of Fluorescence – UF/mg protein and was expressed as percentage of control.

### Total Antioxidant Reactivity (TAR)

TAR, which represents the reactivity of the tissue antioxidants, was determined by measuring the luminol chemiluminescence intensity induced by 2,2′-azo-bis-(2-amidinopropane) (ABAP) [44]. The background chemiluminescence was measured by addicting 2 mM ABAP (in 0.1 M glycine buffer, pH 8.6) into a glass scintillation vial. After, luminol (4 mM) were added to each vial and the chemiluminescence was determined. This was considered to be the basal value. Cell homogenates were then added and the chemiluminescence was measured during 60 s. The addition of trolox (calibration curve) or cells supernatants reduce the chemiluminescence and this rapid reduction in luminol intensity is considered measure of TAR capacity. The ratio between the initial and the final chemiluminescente values was used to calculate TAR. TAR values were calculated as nmol trolox/mg protein and are presented as percentage of control.

### Mitochondrial Membrane Potencial (JC-1 assay)

For determination of the mitochondrial membrane potential, after resveratrol and H_2_O_2_ treatments, cells were incubated for 0.5 h with JC-1 (5,5′,6,6′-tetrachloro-1,1′,3,3′-tetraethylbenzimidazolylcarbocyanine iodide, 2 µg/ml). After, cells were centrifuged, washed once with HBSS and transferred to a 96-well plate and fluorescence was measured using excitation and emission wavelengths of 485 and 540/590 nm, respectively. The mitochondrial membrane potential was calculated using the ratio of 590 nm (red fluorescent J-aggregates)/540 nm (green monomeric) [45], [46].

### Creatine Kinase (CK) Activity

Cells were homogenized with a 0.9% saline solution and pre-incubated for 15 min at 37°C in a mixture containing: 7 mM phosphocreatine, 9 mM MgSO_4_, 60 mM Tris–HCl buffer (pH 7.5). Incubation was started by the addition of 3.2 mM ADP plus 0.8 mM reduced glutathione. The reaction was stopped after 10 min by the addition of 10 µmol *p*-hydroxymercuribenzoic acid. The reagent concentrations and the incubation time were chosen to assure linearity of the enzymatic reaction. Appropriate controls were carried out to measure chemical hydrolysis of phosphocreatine. The creatine formed was estimated according to the colorimetric method [47]. The color was developed by the addition of 20% *α*-naphthol and 20% diacetyl and read after 20 min at 540 nm. Results were obtained as *µ*mol of creatine formed/min/mg protein.

### Superoxide Dismutase (SOD) Activity

SOD activity was determined using the RANSOD kit from Randox (Autrim, UK). The method is based on the formation of red formazan from the reaction of 2-(4-iodophenyl)-3-(4-nitrophenol)-5-phenyltetrazolium chloride and superoxide radical produced in the incubation medium from the xanthine and xanthine oxidase reaction system, which is assayed spectrophotometrically at 505 nm. The inhibition of the produced chromogen is proportional to the activity of the SOD present in the sample. A 50% inhibition is defined as one unit of SOD and the specific activity is represented as U/mg protein.

### Catalase (CAT) Activity

CAT activity was assayed by the method of Aebi [48], by measuring the absorbance decrease at 240 nm in a reaction medium containing 20 mM H_2_O_2_, 0.1% Triton X-100, 10 mM potassium phosphate buffer, pH 7.0 and 50 µg protein. One unit (U) of the enzyme is defined as 1 µmol of H_2_O_2_ consumed per minute and the specific activity is reported as U/mg protein.

### Glutathione Peroxidase (GPx) Activity

GPx activity was measured by the method of Wendel [49], using *tert*-butyl-hidroperoxide as substrate. GPx activity was determined by monitoring NADPH (0.1 mM) disappearance at 340 nm in a medium containing 2 mM GSH, 0.15 U/ml glutathione reductase, 0.4 mM azide and 0.5 mM *tert*-butyl-hidroperoxide. One GPx unit is defined as 1 µmol of NADPH consumed per minute and the specific activity is represented as U/mg protein.

### Extracellular Glutathione (GSH)

Extracellular GSH levels were measured according to Browne and Armstrong with modifications [17], [50]. We also examined the effect of HO1 inhibitor on extracellular GSH levels, incubating Zinc Protoporphyrin IX (ZnPP IX) 10 µM, for 0.5 h before the treatment described for resveratrol and H_2_O_2_. Extracellular medium were diluted in 100 mM sodium phosphate buffer, pH 8.0, containing 5 mM EDTA, and protein was precipitated with 1.7% meta-phosphoric acid. After centrifugation, supernatant was assayed with *o*-phthaldialdeyde (1 mg/mL methanol) at room temperature for 15 min. Fluorescence was measured using excitation and emission wavelengths of 350 and 420 nm, respectively. A calibration curve was performed with standard GSH (0–500 µM) and the concentrations were calculated as nmol/mg of protein. The results were expressed as percentage of control.

### Tumor Necrosis Factor α (TNFα) Levels

This assay was carried out in extracellular medium, using a rat TNFα ELISA from PeproTech (USA). To test whether the effect of resveratrol against H_2_O_2_-induced TNFα levels was through HO1, we used a specific inhibitor, Zinc Protoporphyrin IX (ZnPP IX) 10 µM, for 0.5 h before the treatment described for resveratrol and H_2_O_2_.

### Protein Determination

Protein content was measured by Lowry’s method using bovine serum albumin as standard [51].

### Statistical Analysis

All quantitative data and experiments described in this study were repeated at least three times. Results are expressed as mean ± S.E.M. Differences between groups were analyzed statistically by two way analysis of variance (ANOVA) followed by the Tukeýs test using SPSS (Statistical Package for the Social Sciences software, version 16.0 for Windows). Values of *P*<0.05 were considered statistically significant.

## Results

### Resveratrol Decreased Nitrite Levels

**Figure 1:**
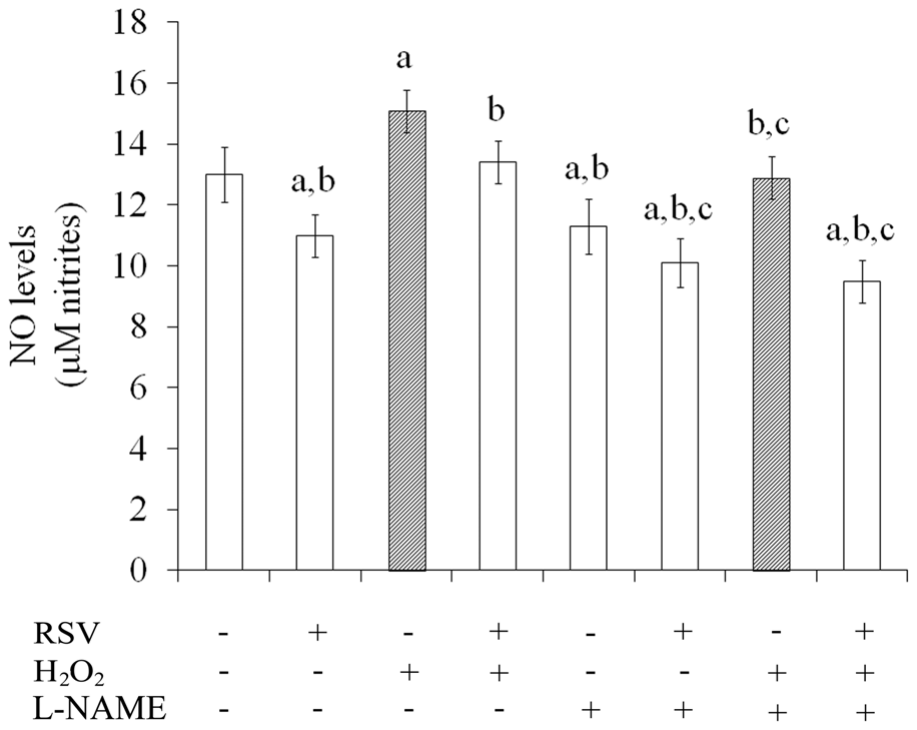
Resveratrol decreased nitrite production. Cells were pre-treated for 1 h with 100 µM resveratrol (RSV) followed by the addition of 1 mM H_2_O_2_ for 0.5 h. Nitrite production was measured as described in the Experimental procedures section. Data represent mean ± S.E.M of three independent experimental determinations performed in triplicate, analyzed statistically by two-way ANOVA followed by the Tukey’s test. (a) indicates significant differences from control (P<0.05). (b) indicates significant differences from H_2_O_2_ (P<0.05). (c) indicates significant differences from L-NAME (P<0.05).

The production of NO was indirectly measured by the formation of nitrite, expressed in µM. Resveratrol *per se* decreased nitrite production, from 13±0.9 to 11±0.7 µM, compared to the control conditions (Fig. 1). H_2_O_2_ increased nitrite formation up to 16% (from 13±0.9 to 15.1±0.7 µM). This effect was completely prevented by resveratrol. To test whether decreased in nitrite accumulation induced by resveratrol was dependent on NOS activity, we examined the effect of resveratrol in the presence of the L-NAME (N^ω^-nitro-L-arginine methyl ester), a NOS inhibitor. The co-incubation of resveratrol with L-NAME decreased the nitrite levels up to 22% compared to the basal value (Fig. 1).

### Resveratrol Prevented H_2_O_2_-induced iNOS Expression

**Figure 2:**
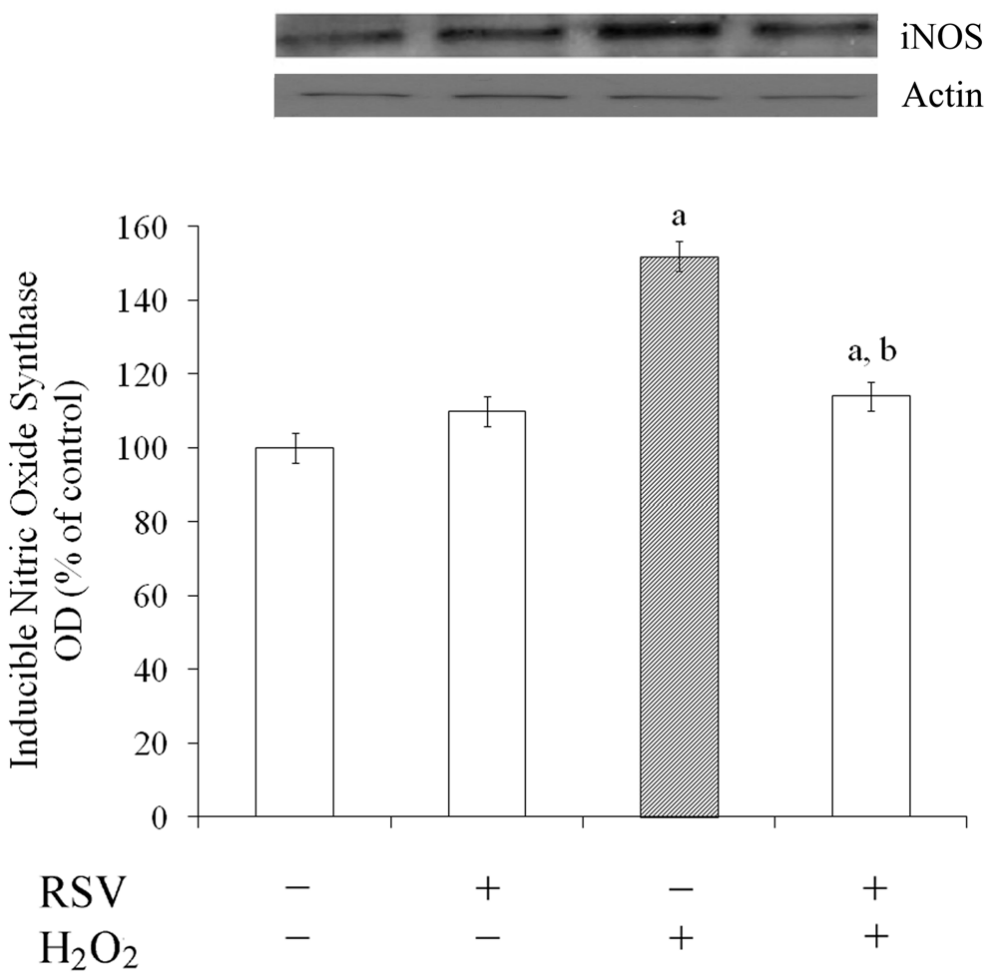
Effects of resveratrol on iNOS expression. Cells were pre-treated for 1 h with 100 µM resveratrol (RSV) followed by the addition of 1 mM H_2_O_2_ for 0.5 h. Western blot for iNOS was performed as described in the Experimental procedures section. Data are expressed as percentage of control value (assumed to be 100%) and represent the optical density (OD) of mean ± S.E.M of three independent experimental determinations performed in triplicate, analyzed statistically by two-way ANOVA followed by the Tukey’s test. (a) indicates significant differences from control (P<0.01). (b) indicates significant differences from H_2_O_2_ (P<0.01).

Considering that the excess production of NO, generated primarily by iNOS, has been implicated as a mediator of cellular injury, we aimed to determine the effect of resveratrol on iNOS expression in the presence of H_2_O_2_. As expected, H_2_O_2_ increased iNOS immunocontent (about 50%) compared with the control conditions (Fig. 2). This effect was inhibited by resveratrol to values near to basal levels, indicating that resveratrol could effectively suppress iNOS expression, which was stimulated by H_2_O_2_.

### Resveratrol Enhanced HO1 Expression

**Figure 3:**
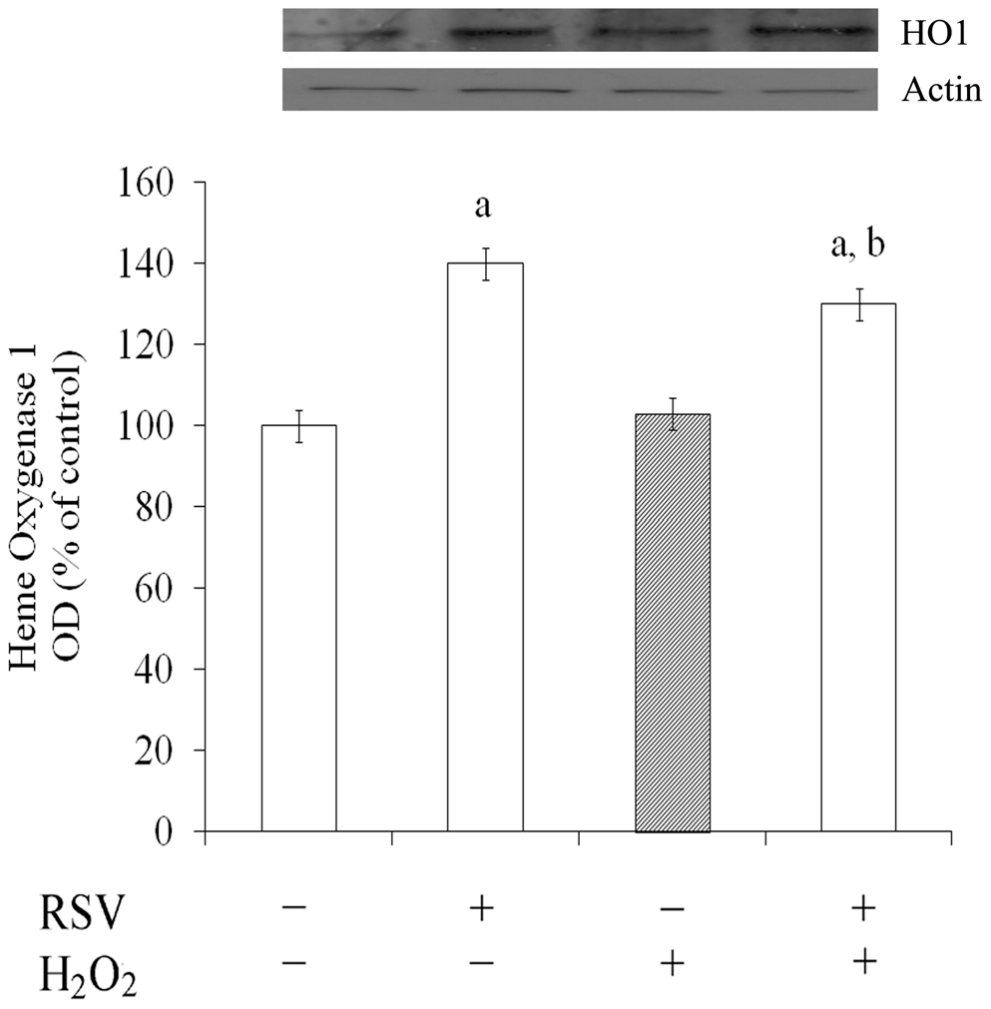
Resveratrol increased HO1 expression. Cells were pre-treated for 1 h with 100 µM resveratrol (RSV) followed by the addition of 1 mM H_2_O_2_ for 0.5 h. Western blot for HO1 was performed as described in the Experimental procedures section. Data are expressed as percentage of control value (assumed to be 100%) and represent the optical density (OD) of mean ± S.E.M of three independent experimental determinations performed in triplicate, analyzed statistically by two-way ANOVA followed by the Tukey’s test. (a) indicates significant differences from control (P<0.01). (b) indicates significant differences from H_2_O_2_ (P<0.01).

Increases in HO1 protein levels are associated to protection against stress conditions, such as oxidative stress, hypoxia and neurodegenerative disorders [7]. Then we evaluated the immunocontent of this enzyme. Fig. 3 shows that resveratrol *per se* and under oxidative insult was able to increase the expression of HO1 (by about 40% and 30%, respectively) in C6 astrocyte cell line. H_2_O_2_ did not affect the HO1 expression.

### Resveratrol Reduced Intracellular ROS Production through HO1

**Figure 4:**
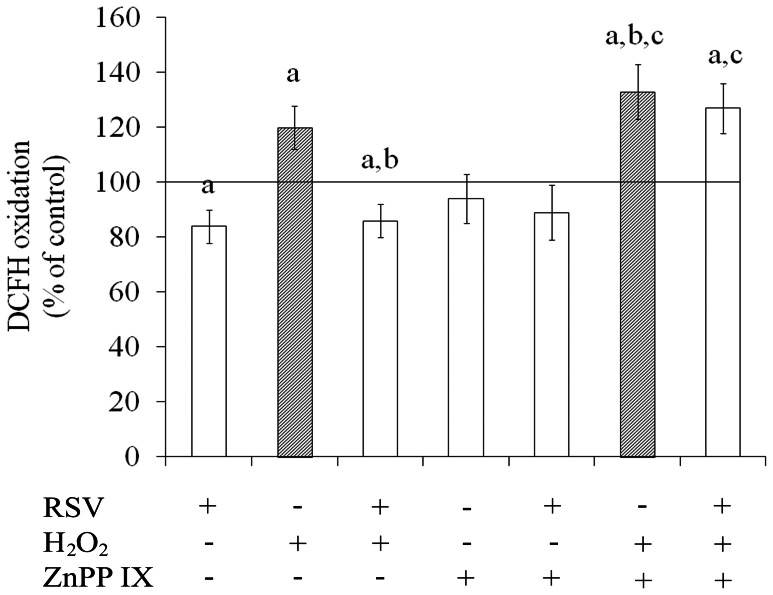
Effects of resveratrol on intracellular ROS production. Cells were pre-treated for 1 h with 100 µM resveratrol (RSV) followed by the addition of 1 mM H_2_O_2_ for 0.5 h. Cells were also pre-incubated for 0.5 h with ZnPP IX (10 µM), a HO1 inhibitor, before the pre-treatment with resveratrol. Intracellular ROS production was measured as described in the Experimental procedures section. Data are expressed as percentage of control value and represent mean ± S.E.M of three independent experimental determinations performed in triplicate, analyzed statistically by two-way ANOVA followed by the Tukey’s test. (a) indicates significant differences from control (P<0.05). (b) indicates significant differences from H_2_O_2_ (P<0.05) and (c) indicates significant differences from ZnPP IX inhibitor (P<0.05).

As an imbalance between production and removal of ROS toward the pro-oxidative state can be promoted by the cellular exposure to H_2_O_2_, we investigated the effect of resveratrol and resveratrol combined with HO1 inhibitor on intracellular ROS production by DCFH oxidation method (Fig. 4). Resveratrol *per se* decreased by about 16% the DCFH oxidation compared to control conditions. In addition, resveratrol was able to prevent the increase of ROS production induced by H_2_O_2_. When cells were pre-incubated with ZnPP IX (10 µM) resveratrol partially decreased DCFH oxidation and did not prevent the effect of H_2_O_2_ exposure, indicating that antioxidant effect of resveratrol probably was through HO1. Interestingly, under HO1 inhibitor, H_2_O_2_ potentiated the increase in ROS levels.

### Resveratrol Increased Total Antioxidant Reactivity (TAR)

**Figure 5:**
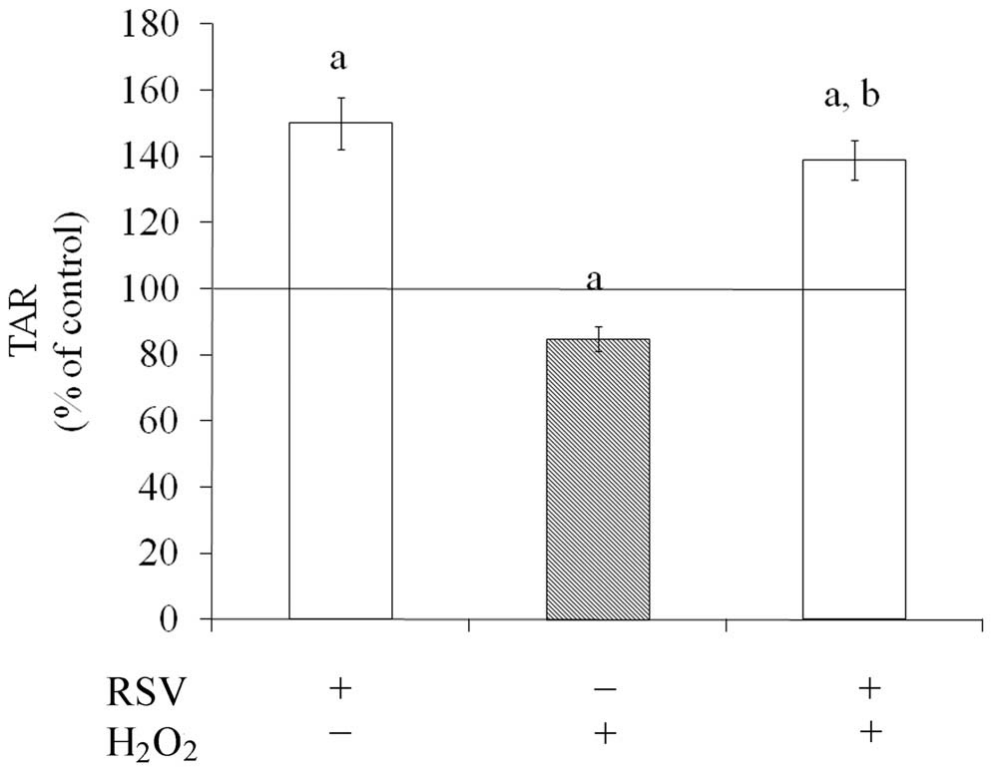
Effects of resveratrol on TAR levels. Cells were pre-treated for 1 h with 100 µM resveratrol (RSV). After pre-treatment, cells were exposed to 1 mM H_2_O_2_ for 0.5 h. TAR levels were measured as described in the Experimental procedures section. The basal TAR levels, assumed to be 100%, are indicated by the line. Each value is the mean ± S.E.M of three independent experimental determinations performed in triplicate, analyzed statistically by two-way ANOVA followed by the Tukey’s test. (a) indicates significant differences from control (P<0.001). (b) indicates significant differences from H_2_O_2_ (P<0.001).

Resveratrol increased (50%) TAR levels compared to control conditions (Fig. 5). Under oxidative insult the TAR levels were reduced by about 15% compared to basal value. This effect was completely prevented by resveratrol, which increased TAR levels up to 55%, compared to H_2_O_2_-oxidative insult.

### Resveratrol Prevented Mitochondrial Dysfunction

**Figure 6:**
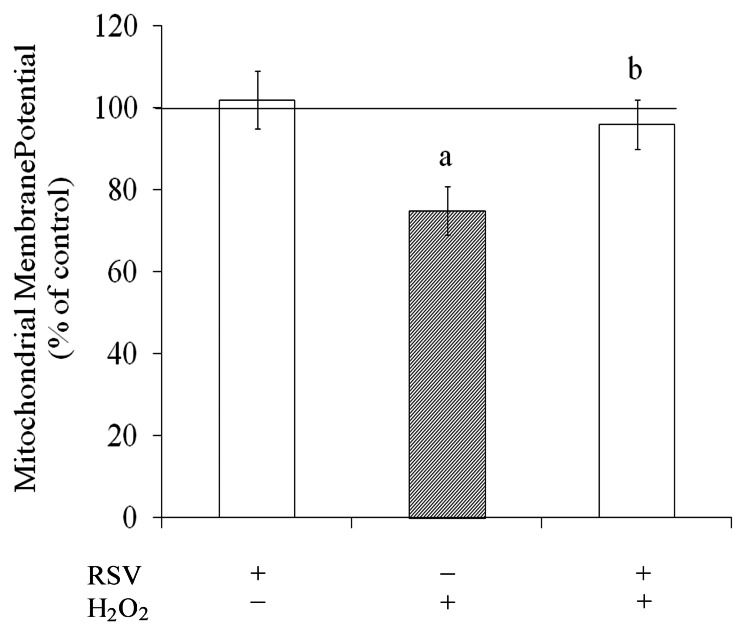
Resveratrol prevented mitochondrial dysfunction. Cells were pre-treated for 1 h with 100 µM resveratrol (RSV). After pre-treatment, cells were exposed to 1 mM H_2_O_2_ for 0.5 h. Mitochondrial membrane potential was measured as described in the Experimental procedures section. The control conditions, assumed to be 100%, are indicated by the line. Each value [the ratio of 590 nm (red fluorescent J-aggregates)/540 nm (green monomeric)] is the mean ± S.E.M of three independent experimental determinations performed in triplicate, analyzed statistically by two-way ANOVA followed by the Tukey’s test. (a) indicates significant differences from control (P<0.05). (b) indicates significant differences from H_2_O_2_ (P<0.05).

The mitochondria have emerged as key regulators of oxidative stress and cytotoxicity [32], [52]. As expected H_2_O_2_ reduced the mitochondrial membrane potential by about 25%, inducing mitochondrial dysfunction and resveratrol completely prevented this effect (Fig. 6). Resveratrol *per se* did not affect the mitochondrial membrane potential.

### Resveratrol Prevented the Impairment in CK Activity Induced by H_2_O_2_

**Figure 7:**
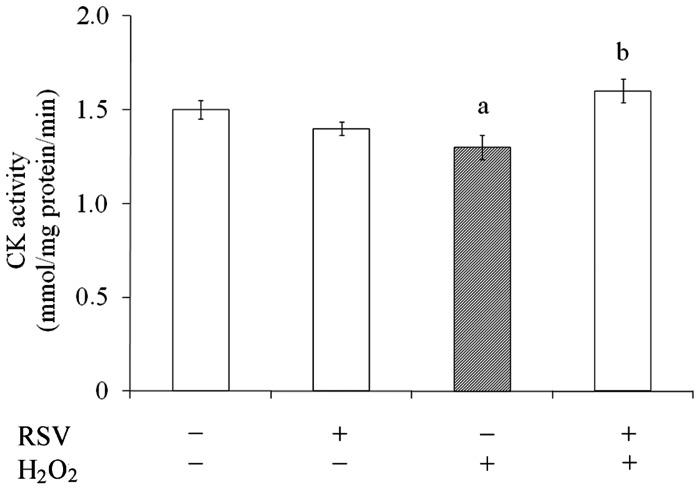
Effects of resveratrol on CK activity. Cells were pre-treated for 1 h with 100 µM resveratrol (RSV). After pre-treatment, cells were exposed to 1 mM H_2_O_2_ for 0.5 h. CK activity was measured as described in the Experimental procedures section. Data represent mean ± S.E.M of three independent experimental determinations performed in triplicate, analyzed statistically by two-way ANOVA followed by the Tukey’s test. (a) indicates significant differences from control (P<0.05). (b) indicates significant differences from H_2_O_2_ (P<0.05).

The enzyme CK is a target of ROS/RNS and we evaluated the effect of resveratrol on the CK activity in the presence or absence of H_2_O_2_ (Fig. 7). As expected, oxidative insult decreased CK activity by 15% (from 1.5±0.1 to 1.3±0.1) and resveratrol was able to completely prevent this effect. Resveratrol *per se* did not affect CK activity.

### Effects of Resveratrol on Superoxide Dismutase (SOD), Catalase (CAT) and Glutathione Peroxidase (GPx) Activity

**Figure 8:**
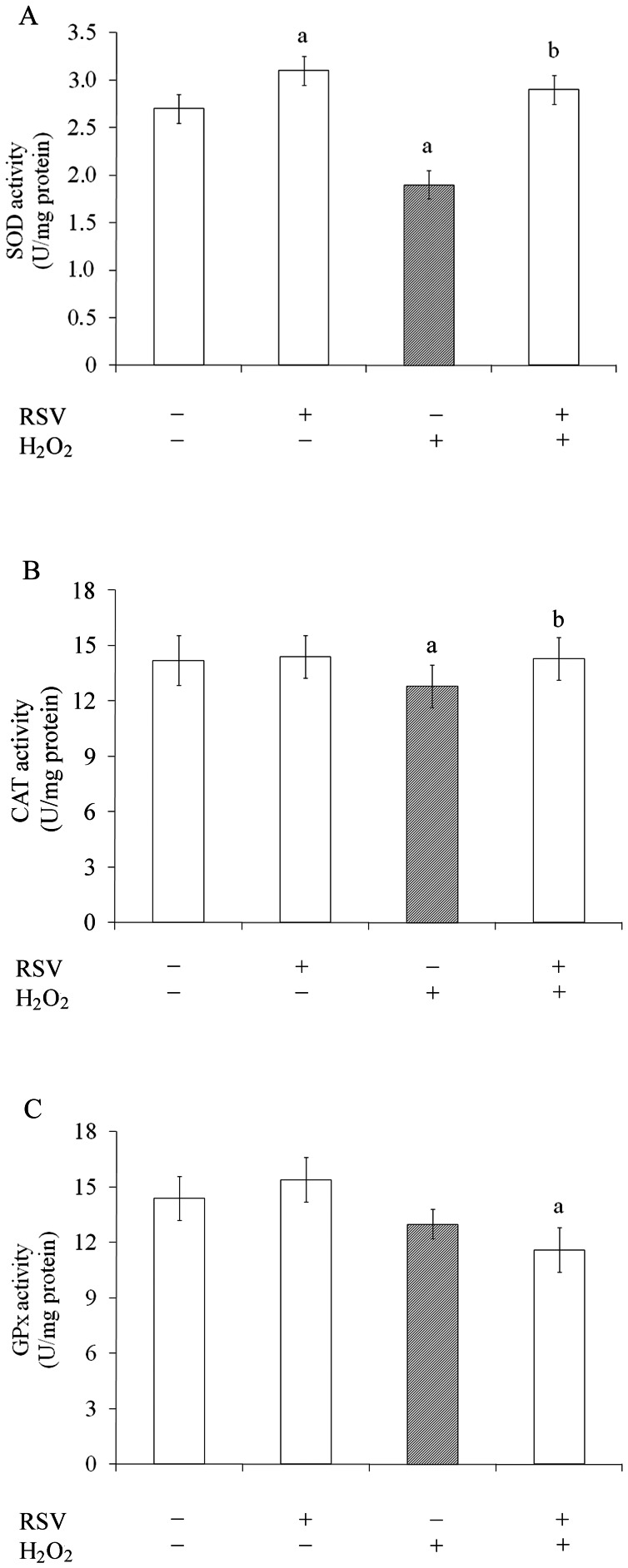
Effects of resveratrol on SOD, CAT and GPx activity. Cells were pre-treated for 1 h in the presence of 100 µM resveratrol (RSV) followed by the addition of 1 mM H_2_O_2_ for 0.5 h. SOD (**A**), CAT (**B**) and GPx (**C**) activities were measured as described in the Experimental procedures section. All data represent mean ± S.E.M. of three independent experimental determinations performed in triplicate, analyzed statistically by two-way ANOVA followed by the Tukey’s test. (a) indicates significant differences from control (P<0.05). (b) indicates significant differences from H_2_O_2_ (P<0.05).

In order to determine the effect of resveratrol on antioxidant cellular defenses, we studied the activities of SOD, CAT and GPx (Fig. 8). Resveratrol was able to increase only the activity of SOD (from 2.7±0.1 to 3.1±0.1 U/mg protein) compared to control conditions (Fig. 8A). The H_2_O_2_-induced oxidative stress decreased SOD (to 1.9±0.1 U/mg protein) (Fig. 8A) and CAT (from 14.2±1.3 to 12.8±1.1 U/mg protein) (Fig. 8B) activities, and these effects were prevented by resveratrol. Resveratrol and H_2_O_2_ did not affect GPx activity (Fig. 8C). However, resveratrol decreased GPx activity (20%) under H_2_O_2_ exposure.

### Resveratrol Increased Extracellular GSH Levels

Resveratrol *per se* is able to modulate GSH, the main antioxidant of CNS, increasing intracellular GSH content in C6 cells [17]. Here, we demonstrated that resveratrol increased extracellular GSH levels, by about 13%, compared to the control conditions (Table 1). Resveratrol also increased GSH extracellular content after the H_2_O_2_ exposure. To verify the effect of resveratrol on extracellular GSH was dependent of HO1, we pre-treated the cells with ZnPP IX. HO1 inhibitor prevented the increase of extracellular GSH induced by resveratrol.

**Table 1:** Effect of resveratrol on extracellular GSH content and TNFα levels.

Treatments	Extracellular GSH	TNFα
RSV	113±6 (a)	101±10
H_2_O_2_	90±10	130±10 (a)
RSV+H_2_O_2_	111±7 (a, b)	99±8 (b)
ZnPP IX	98±6	98±9 (b)
RSV+ZnPP IX	102±8 (b)	97±8 (b)
H_2_O_2_+ ZnPP IX	88±10 (a)	127±12 (a, c)
RSV+H_2_O_2_+ ZnPP IX	87±8 (a, c)	125±12 (a, c)

Cells were pre-treated for 1 h in the presence of 100 µM resveratrol (RSV) followed by the addition of 1 mM H_2_O_2_ for 0.5 h. Cells were also pre-incubated for 0.5 h with ZnPP IX (10 µM), a HO1 inhibitor, before the pre-treatment with resveratrol. Extracellular GSH and TNFα levels were measured as described in the Experimental procedures section. Data are expressed as percentage of control value (assumed to be 100%) and represent mean ± S.E.M. of three experimental determinations performed in triplicate, analyzed statistically by two-way ANOVA followed by the Tukey’s test. (a) indicates significant differences from control (P<0.05). (b) indicates significant differences from H_2_O_2_ (P<0.05) and (c) indicates significant differences from ZnPP IX inhibitor (P<0.05).

### Resveratrol Prevented the H_2_O_2_-stimulated TNFα Levels

To determine whether resveratrol could modulate the proinflammatory cytokines under H_2_O_2_ exposure, we measured the TNFα levels. Resveratrol *per se* did not affect the levels of TNFα, but was able to completely prevent the augment induced by H_2_O_2_ exposure, decreasing the levels of this cytokine, from 130±10 to 99±8 (Table 1). We examined whether the effect of resveratrol was dependent of HO1 and pre-treated the cells with HO1 inhibitor, which blocked the effect of resveratrol on H_2_O_2_-stimulated TNFα levels.

## Discussion

The effect of resveratrol in the brain has been studied in a variety of pathological events, including carcinogenesis [53], ischemic injury [54] and neurodegenerative disorders [15], [16], [55]. However, the cellular mechanisms underlying resveratrol-induced selective protection to the neural system needs to be better elucidated. The present data shows that resveratrol is able to modulate important antioxidant defenses in C6 astrocyte cell line.

High concentrations of H_2_O_2_ induce oxidative-nitrosative stress, which can lead to lipid, protein and DNA oxidation, causing cellular damage [56], [57]. It is important to emphasize that in this study H_2_O_2_ did not induce cell death, in agreement with other studies of our group [17], [41], [58]. Resveratrol decreased NO levels and RNS (possibly peroxynitrite) implicated in the pathogenesis of neurodegenerative disorders like Alzheimer’s and Parkinson’s diseases [34]. Peroxynitrite, resulted by the reaction between NO and superoxide anion, is one of the main molecules responsible for the cellular damage in neurodegenerative disorders [34].

Oxidative stress upregulates the expression of iNOS and presumably would lead to increase in the NO concentrations [16]. NO is synthesized during the stoichiometric conversion of L-arginine to L-citrulline in the presence of oxygen and nicotinamide adenine dinucleotide phosphate (NADPH), which is catalyzed by NOS [59]. Our results have indicated that resveratrol decreases iNOS expression. Previous works already demonstrated that resveratrol may act through iNOS [16], [60], [61]. Thus, these results suggest that resveratrol may have a protective effect against diseases associated with increase in iNOS synthesis and NO levels.

Resveratrol *per se* was also able to modulate the activity of HO1. Recently, Sakata et al, 2010 [30] showed that HO1 might be a unique candidate by which resveratrol can induce an endogenous cellular pathway which leads to building cellular and/or organ resistance to stress, indicating a neuroprotective effect. In agreement with Sakata, we showed that resveratrol, via HO1, has antioxidant effects and thus could explain the actions of resveratrol in CNS [7]. Furthermore, there is a relationship between HO1 and iNOS. The nuclear factor-erythroid-2-related factor 2 (Nrf2) regulates the transcription of HO1, which acts as a scavenger of NO [31]. Nrf2 also mediates neuroprotection and modulates several detoxification genes that encode antioxidant proteins, such as GSH system and thioredoxin, regulators of intracellular redox environment [61], [62], [63], [64]. Resveratrol activates Nrf2 and all these regulators [61], [64], [65], [66], [67], [68], [69], [70]. Excess of NO acts as a positive signal to increase HO1, which in turn, is able to scavenge NO and block the activity of iNOS, to prevent further NO production. Resveratrol, under oxidative conditions, induced a decrease in iNOS and an increase in HO1 immunocontent. Thus, HO1 can also be critical to signaling antioxidant response of resveratrol.

Besides, resveratrol decreased DCFH oxidation and prevented H_2_O_2_-oxidative insult. This suggests strong antioxidant properties for this polyphenol [71]. Excessive free radical, such as peroxynitrite, can lead to lipid, protein and DNA oxidation; and these events are associated to age-related diseases [16], [72]. Our group has been demonstrated that resveratrol modulates glutamate metabolism and protects against genotoxicity, probably by their scavenger properties and astrocyte activation [17], [41]. Resveratrol under oxidative insult inhibited iNOS and decreased DCFH oxidation, emerging as an important molecule which provides protection against cellular toxicity. Our data on intracellular ROS production also indicated the involvement of HO1 signaling pathway in the protective mechanism of resveratrol against H_2_O_2_-induced oxidative stress.

TAR levels represent the antioxidant capacity of cells and resveratrol was able to increase TAR levels by about 50%. This suggests that resveratrol displays antioxidant effects, protecting cells against oxidative damage. It might avoid free radical generation and improve antioxidant defenses, such as GSH [73], [74], [75]. H_2_O_2_ decreased TAR levels. This effect elicited by resveratrol has been mainly attributed to its intrinsic antioxidant properties. However, it is important to mention that it is also able to modulate diverse cell activities independently of its antioxidant properties [76].

The mitochondrial membrane potential has been implicated as a factor in impaired mitochondrial function [77]. The impairment in this organelle have been shown to occur in various models of cell injury [32]. A decrease in mitochondrial membrane potential following an intense production of ROS and RNS induces mitochondrial disruption, inhibition of mitochondrial respiratory chain, reduction in ATP synthesis and cell death [78]. In this sense, in our study H_2_O_2_ generates high levels of ROS/RNS that induce mitochondrial membrane potential dysfunction. Resveratrol totally prevent this damage. In addition, brain cells contain antioxidative defense mechanisms that can protect against oxidative damage and resveratrol may be a key regulator of these defenses.

We also showed that although resveratrol did not modify CK activity *per se*, it was able to prevent H_2_O_2_-induced CK activity decrease. CK consists of a family of enzymes involved in high-energy consuming tissues such as brain and skeletal muscle and it is also very sensitive to oxidative damage (oxidation and nitration). Considering that CK contains in its active site sulphydryl group extremely important for their full operation, it is proposed that reduced activity is related to increase in ROS/RNS production induced by H_2_O_2_, which could oxidize thiol groups, contributing to CK activity inhibition [79]. The marked reduction of CK activity is reported in the brain of patients with oxidative-stress linked neurodegenerative diseases, such as Alzheimer’s and Parkinson’s diseases [79]. This indicates that resveratrol can display *in vivo* function on CK activity in pathologies related to redox imbalance.

The levels of free radicals can be determined by the balance between their rate of production and clearance by various antioxidant compounds and enzymes such as SOD, CAT and GPx. Resveratrol prevented completely the H_2_O_2_-effect reestablishing SOD activity, which in turn can decrease superoxide radicals [80], [81]. In this context, modulation of SOD activity by resveratrol is very important to the neuroprotective effect associated to oxidative stress. However, we also observed an increase in CAT activity compared to oxidative insult, showing that the H_2_O_2_ is decomposed to water [26], [82]. Resveratrol decreases the GPx activity under H_2_O_2_-induced oxidative stress, improving the levels of GSH, the major brain antioxidant, produced by astrocytes. As many redox-active compounds modulate major enzymatic defenses against free radical action [83], [84], [85], [86], the anti- or pro-oxidant role is determined by the set of their actions and cell types studied [5], [35].

We have been showed that resveratrol modulates astroglial parameters related to glutamate metabolism [14], [17], [42] including the increase in glutamate uptake (probably by increase in EAAC1 expression) and GSH levels. GSH is secreted by astrocytes and serves as a substrate to neuronal GSH synthesis [80], [87]. Metal chelators, SOD and antioxidants (*e.g.* resveratrol) inhibit the oxidation of GSH, when it is secreted by astrocytes [80]. Moreover, the increase in GSH levels in glial cells confers neuroprotection in neurodegenerative diseases, such as Alzheimer’s and Parkinson’s diseases [27], [88]. In this sense, resveratrol increases extracellular GSH levels. The effect of resveratrol on extracellular GSH probably involves the HO1 pathway. In addition, Escartin et al, 2011 [89] reported that Nrf2, the upregulator of HO1, facilitates the glutathione synthesis by regulation of EAAC1, the main glutamate transporter present in C6 astroglial cells. The increase in GSH biosynthesis through Nrf2 also protected glial cells against oxidative damage [90].

Lee et al, 2010 [88] demonstrated that a depletion of GSH in glial cells induces inflammatory response. TNFα plays important roles in ROS production [91] and it also potentiates NO production in astrocytes [92]. Thus, an extracellular stimulus such as H_2_O_2_ induces an increase in ROS/RNS production and in TNFα levels. In this sense, resveratrol was able to prevent the proinflammatory cytokine increase induced by H_2_O_2_ exposure, probably by HO1 pathway, an upstream signal transduction of nuclear factor-κB (NF-κB) and iNOS expression [31]. Even though its mechanism of action remains not fully understood, resveratrol may exert its effects by antioxidant/scavenger activity, by modulation of NO metabolism and HO1 expression or by anti-inflammatory effect [6], [16], [29], [30], [93], [94], [95], [96].

**Figure 9:**
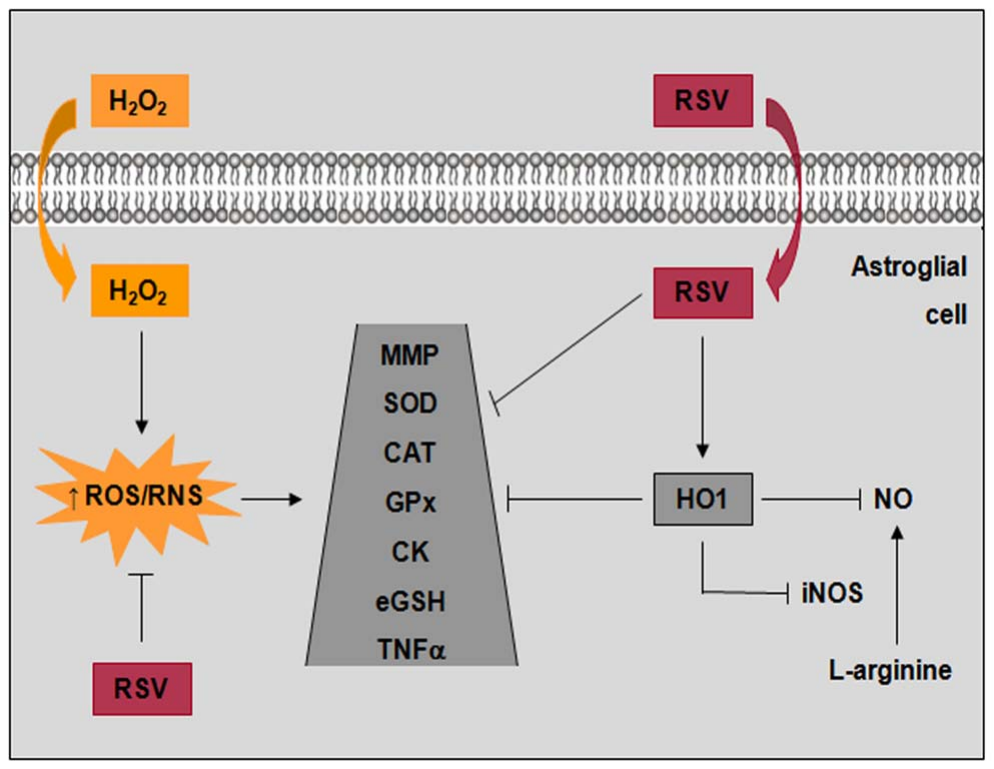
Schematic illustration of the mechanism underlying the protective effect of resveratrol against H_2_O_2_-induced oxidative stress in C6 astrocyte cell line. H_2_O_2_ exposure induces increase in ROS/RNS levels, which are prevented by resveratrol (RSV). Oxidative-nitrosative stress induces impairment in mitochondrial membrane potential (referred in the figure as MMP), SOD, CAT, GPx, CK, extracellular GSH (referred in the figure as eGSH) and TNFα. RSV enhances HO1 expression, followed by decreased iNOS expression and consequently NO levels attenuation. Thus, resveratrol is able to confer protection against H_2_O_2_-induced oxidative stress in C6 astrocyte cell line, probably, via HO1 activation, antioxidant/scavenger activity and/or anti-inflammatory effect.

In summary, we demonstrated the cytoprotective effects of resveratrol against oxidative damage in C6 astrocyte cell line. The main conclusions of this study are depicted in Fig. 9, which displays that resveratrol strongly prevented the increase in ROS/RNS production, iNOS expression and TNFα levels induced by H_2_O_2_ in C6 astrocyte cell line and the putative involvement of HO1 activation in the glioprotective role of resveratrol. H_2_O_2_ exposure induced decrease in mitochondrial membrane potential, TAR levels, SOD, CAT and CK activity and resveratrol was also able to protect glial cells. Moreover, resveratrol *per se* increased the HO1 expression, the TAR levels, SOD activity and extracellular GSH content and decreased the basal levels of ROS/RNS. Our results suggest that resveratrol modulates important functions related to the maintenance of the cellular redox environment through HO1 signaling pathway. Overall, these observations suggest that resveratrol may potentially be useful for therapeutic purposes as a potent inducer of HO1 for the protection of glial cells against oxidative response.
